# Stereotactic radiotherapy of intrapulmonary lesions: comparison of different dose calculation algorithms for Oncentra MasterPlan®

**DOI:** 10.1186/s13014-015-0354-3

**Published:** 2015-02-22

**Authors:** Almut Troeller, Sylvia Garny, Sophia Pachmann, Steffi Kantz, Sabine Gerum, Farkhad Manapov, Ute Ganswindt, Claus Belka, Matthias Söhn

**Affiliations:** Department of Radiation Oncology, University of Munich, Marchioninistr. 15, 81377 Munich, Germany

**Keywords:** Lung SBRT, Dose calculation algorithms, Collapsed Cone, Pencil Beam, Small pulmonary lesions

## Abstract

**Background:**

The use of high accuracy dose calculation algorithms, such as Monte Carlo (MC) and Collapsed Cone (CC) determine dose in inhomogeneous tissue more accurately than pencil beam (PB) algorithms. However, prescription protocols based on clinical experience with PB are often used for treatment plans calculated with CC. This may lead to treatment plans with changes in field size (FS) and changes in dose to organs at risk (OAR), especially for small tumor volumes in lung tissue treated with SABR.

**Methods:**

We re-evaluated 17 3D-conformal treatment plans for small intrapulmonary lesions with a prescription of 60 Gy in fractions of 7.5 Gy to the 80% isodose. All treatment plans were initially calculated in Oncentra MasterPlan® using a PB algorithm and recalculated with CC (CC_re-calc_). Furthermore, a CC-based plan with coverage similar to the PB plan (CC_cov_) and a CC plan with relaxed coverage criteria (CC_clin_), were created. The plans were analyzed in terms of D_mean_, D_min_, D_max_ and coverage for GTV, PTV and ITV. Changes in mean lung dose (MLD), V_10Gy_ and V_20Gy_ were evaluated for the lungs. The re-planned CC plans were compared to the original PB plans regarding changes in total monitor units (MU) and average FS.

**Results:**

When PB plans were recalculated with CC, the average V_60Gy_ of GTV, ITV and PTV decreased by 13.2%, 19.9% and 41.4%, respectively. Average D_mean_ decreased by 9% (GTV), 11.6% (ITV) and 14.2% (PTV). D_min_ decreased by 18.5% (GTV), 21.3% (ITV) and 17.5% (PTV). D_max_ declined by 7.5%. PTV coverage correlated with PTV volume (p < 0.001). MLD, V_10Gy_, and V_20Gy_ were significantly reduced in the CC plans. Both, CC_cov_ and CC_clin_ had significantly increased MUs and FS compared to PB.

**Conclusions:**

Recalculation of PB plans for small lung lesions with CC showed a strong decline in dose and coverage in GTV, ITV and PTV, and declined dose in the lung. Thus, switching from a PB algorithm to CC, while aiming to obtain similar target coverage, can be associated with application of more MU and extension of radiotherapy fields, causing greater OAR exposition.

## Background

The use of high accuracy dose calculation algorithms, such as Monte Carlo (MC) and Collapsed Cone (CC) has increased during the past few years due to increased speed of the algorithms and availability of advanced computational technology. These algorithms are capable of estimating dose in inhomogeneous media more accurately than simpler dose calculation algorithms of pencil beam (PB) type. It is well known that the pencil beam algorithms lack accurate modeling of lateral scatter and -backscatter, because they neglect tissue density inhomogeneity in directions other than the beam and assume water density instead. In contrast, CC and MC include more accurate inhomogeneity corrections [1]. MC simulations, which explicitly model particle interactions and -transport in the patient, are generally considered the gold standard of dose calculation. A popular choice for dose calculation are CC based algorithms that approximately model lateral scattering and -backscatter via density dependent anisotropic rescaling of pre-calculated point kernels for effects from secondary particle transport. In the following, we investigate dose calculation differences between PB and CC, which are the two dose calculation algorithms choices implemented in our clinical 3D-CRT treatment planning system (TPS) Oncentra MasterPlan® (OTP, Version 4.2, Nucletron).

Differences in dose calculation accuracy in the patient between the different algorithms can be expected to be most predominant in anatomic regions with large local tissue density inhomogeneities and density discontinuities, such as small lung lesions surrounded by low density lung tissue. When stereotactic radiotherapy of intrapulmonary lesions was first introduced into routine clinical treatment, most institutions calculated dose distributions using dose calculation programs based on PB type algorithms. However, it is now widely and clinically accepted that dose calculation algorithms of the PB type generally do not provide enough accuracy for dose calculation in inhomogeneous tissue [2]. Recent recommendations for the implementation of stereotactic ablative radiotherapy (SABR) of lung cancer therefore include the use of those adapted algorithms. However, historically, prescription schemes were based on clinical experience with PB. Recent guidelines such as the ICRU (ICRU-62, Bethesda, Md, USA, 1999) recommend similarly strict requirements for treatment plans calculated with CC or MC. Because of the insufficient modeling of lateral scattering and -backscatter, the dose to the tumor is usually overestimated when using PB and the same plan may indicate lower dose when recalculated with CC [3,4]. This will lead to treatment plans with changes in field size and MUs delivered, as well as changes in dose to organs at risk (OAR) when creating a treatment plan based on a CC dose calculation algorithm without proper adjustment of the prescription.

In order to quantify differences between dose distributions calculated with PB and CC in patients treated at our institution, we examined treatment plans of patients that received SABR of intrapulmonary lesions. These patients usually have smaller tumors and may show large discrepancies between dose calculated with PB and CC. There is a variety of different implementations of PB algorithms, that may differ in how severely calculated dose differs from delivered dose as estimated with CC. In this study we investigate the enhanced PB and CC algorithms used in the Oncentra MasterPlan® treatment planning system.

## Methods

The treatment plans of 17 patients treated for small intrapulmonary lesions at our institution between 2008 and 2010 were retrospectively evaluated (Table 1). For each patient 3 computed tomography (CT) scans with a 3 mm slice gap were acquired prior to treatment: a free-breathing scan, a maximum inhale and a maximum exhale scan. The scans were then imported to the Oncentra MasterPlan® treatment planning system (Version 4.2, Nucletron). The gross tumor volume (GTV) was contoured on each of the CT scans and an internal target volume (ITV) was defined by forming the union of all GTVs. The planning target volume (PTV) was created from the ITV, by adding a 7 mm margin laterally and 9 mm in the cranial-caudal direction. For all patients, a cumulative physical dose of up to 60 Gy was prescribed to the planning target volume (PTV) in single fractions of 7.5 Gy, such that the 80% isodose line completely covered the PTV.

**Table 1:** **Tumor size, volume and location for all patients**

**Patient**	**Maximum tumor diameter (cm)**	**Total volume GTV (ccm)**	**Tumor location**	**Central/peripheral** ^**†**^
1	1.6	2.0	RLL	peripheral
2	1.6	3.5	RUL	central
3	4.1	41.2	RUL	peripheral
4	3.1	12.1	LLL	peripheral
5	2.8	6.4	R	central
6	1.5	1.4	RUL	peripheral
7	1.2	4.6	RLL	peripheral
8	2.5	3.8	R	central
9	1.2	1.2	R	central
10	2.6	15.2	RLL	peripheral
11	3.0	17.0	LUL	central
12	2.5	4.4	RUL	peripheral
13	2.5	27.0	RLL	peripheral
14	3.9	27.9	RLL	peripheral
15	4.6	61.6	RLL	central
16	1.8	4.7	RUL	peripheral
17	4.0	36.2	LUL	peripheral

^†^For the purpose of this study we defined tumors that were directly attached to the mediastinum or pleura as peripheral and all others (fully surrounded by low density lung tissue) as central.

All dose calculations were performed on the free-breathing CT scan without density overrides. The 3D conformal, un-modulated treatment plans were designed for a Siemens Oncor treatment unit with a 10 mm leaf-width multi-leaf collimator (MLC). Mixed photon energy was used (6 and 15 MV) incorporating 7 beams on average. The beam model was verified against experiment and accepted for clinical use.

The dose distributions of the original treatment plans were calculated using OTP’s enhanced pencil beam (PB) algorithm. For the purpose of this study, all plans were recalculated with the “enhanced CC” algorithm implemented in OTP (CC_re-calc_), without altering the field shape, field size, beam setup, or monitor units. Furthermore we created two additional plans calculated with CC for each patient: A plan that resembles what would currently be considered acceptable for a patient in our clinic (CC_clin_) and a stricter second plan (CC_cov_) that obtained coverage similar to the original PB plan. The former constitutes a clinically accepted compromise in our institution that may formally violate the PTV coverage criteria mentioned above in low density parts of the PTV where full dose build-up is difficult to realize for physical reasons. The latter was created to demonstrate how dose to organs at risk would change in the hypothetical scenario of switching from a PB algorithm to a CC algorithm without adapting the criteria for an acceptable plan.

For the CC_clin_ and CC_cov_ plans MUs as well as field size and shape had to be adjusted accordingly. For the CC_cov_ the fields were opened in beam’s eye view (BEV) until coverage was achieved, while for the CC_clin_ the distance between the jaws and the PTV-outline were not to exceed 1 cm in BEV. All four plans per patient were then compared to each other. Parameters used for comparison were volume coverage (V_60Gy_), mean dose (D_mean_), dose received by 99% and 1% of the volume (D_99_ and D_1_), minimum dose (D_min_), and maximum dose (D_max_) for the GTV, ITV and PTV. Estimated coverage in dependence on GTV volume was also considered. Furthermore we evaluated D_mean_, V_20Gy_ and V_10Gy_ to the lungs, D_mean_ to the heart and D_max_ to the spinal cord and esophagus. Both lungs were contoured as one organ including the PTV. The adjusted CC_clin_ and CC_cov_ treatment plans were further analyzed regarding alterations of MU and field size compared to the original PB plan. The influence of tumor position and movement on coverage was briefly investigated. For the purpose of this study we defined tumors that were directly attached to the mediastinum or pleura as peripheral and all others as central. Statistical significance of the differences between parameters calculated with PB and parameters calculated with CC_re-calc_,CC_clin_ and CC_cov_ was determined using the Wilcoxon signed-rank test (p < 0.05 signifies significant difference). Correlation of the difference in target coverage between PB and CC (i.e. V_60 Gy,PB_ – V_60Gy,CC_) with the target ROI volumes was determined using Spearman’s rank correlation coefficient, ρ. The significance of the correlation was verified with Spearman’s rank test, with the null hypothesis H_0_, that ROI volume and difference in coverage are independent and alternative hypothesis H_a_, that they are dependent. Analysis of the parameters and statistical analyses were performed in Mathematica (Wolfram Research, Inc., Mathematica, Version 9.0.1, Champaign, IL (2013)).

## Results and discussion

### Original PB plan vs. CC_re-calc_

For the target structures GTV, ITV and PTV all evaluated parameters (V_60Gy_, D_mean_, D_min_, D_max_, D_99_, D_1_) were significantly lower for the plans that were re-calculated based on the CC algorithm (CC_re-calc_) as opposed to the original ones incorporating the PB algorithm (p < 0.001).

All OAR parameters considered in this study (p < 0.01), except D_max_ to the spinal cord, were statistically significantly lower when the PB plan was recalculated with CC. However, the differences regarding heart and spinal cord were of relatively small magnitude and may not be clinically relevant.

The results for all structures are shown in Table 2.

**Table 2:** **Dose and volume parameters for targets and organs at risk for pencil beam and cc calculations**

**ROI**	**Parameter**	**PB**	**CC**_**re-calc**_	**CC**_**cov**_	**CC**_**clin**_
GTV	V_60Gy_ (%)	99.8 ± 0.9	86.6 ± 26.5	99.6 ± 1.0	99.4 ± 1.5
	D_mean_ (Gy)	73.7 ± 1.6	67.0 ± 4.4	72.8 ± 1.5	72.5 ± 1.6
	D_min_ (Gy)	63.1 ± 11.0	51.4 ± 9.6	61.3 ± 8.5	59.5 ± 9.6
	D_99_ (Gy)	67.9 ± 7.1	56.8 ± 7.6	65.5 ± 5.4	64.2 ± 6.3
	D_max_ (Gy)	76.8 ± 1.4	71.6 ± 4.0	76.6 ± 1.3	76.6 ± 1.3
	D_1_ (Gy)	76.4 ± 1.4	71.3 ± 3.9	76.3 ± 1.3	76.4 ± 1.3
ITV	V_60Gy_ (%)	99.6 ± 1.7	79.8 ± 30.4	98.4 ± 4.3	97.3 ± 5.7
	D_mean_ (Gy)	73.3 ± 1.7	64.8 ± 5.2	71.0 ± 2.1	70.6 ± 2.3
	D_min_ (Gy)	60.6 ± 12.4	47.7 ± 11.0	57.9 ± 11.7	56.3 ± 11.7
	D_99_ (Gy)	66.0 ± 10.5	53.2 ± 9.9	62.1 ± 8.6	60.7 ± 9.4
	D_max_ (Gy)	77.0 ± 1.5	71.6 ± 4.0	76.6 ± 1.3	76.6 ± 1.3
	D_1_ (Gy)	76.7 ± 1.4	71.1 ± 4.0	76.2 ± 1.2	76.2 ± 1.2
PTV	V_60Gy_ (%)	95.1 ± 1.9	55.7 ± 27.0	90.9 ± 12.5	85.5 ± 15.3
	D_mean_ (Gy)	69.9 ± 1.3	60.0 ± 5.7	67.4 ± 2.6	66.6 ± 3.1
	D_min_ (Gy)	43.4 ± 4.9	35.8 ± 3.9	49.2 ± 4.4	46.4 ± 5.2
	D_99_ (Gy)	55.0 ± 1.9	44.3 ± 5.3	56.7 ± 3.7	54.2 ± 4.5
	D_max_ (Gy)	77.0 ± 1.5	71.6 ± 4.0	76.6 ± 1.3	76.6 ± 1.3
	D_1_ (Gy)	76.5 ± 1.4	70.7 ± 4.1	75.8 ± 1.2	75.8 ± 1.2
Lungs	V_20 Gy_ (%)	7.6 ± 4.3	6.8 ± 4.2	9.6 ± 5.0	8.9 ± 4.9
	V_10 Gy_ (%)	14.3 ± 5.7	13.4 ± 6.3	17.4 ± 6.6	16.3 ± 6.5
	D_mean_ (Gy)	5.3 ± 2.1	5.0 ± 2.0	6.6 ± 2.3	6.1 ± 2.3
Heart	D_mean_ (Gy)	3.0 ± 3.5	2.9 ± 3.5	3.6 ± 3.5	3.4 ± 3.5
Spinal cord	D_max_ (Gy)	8.3 ± 8.8	8.1 ± 8.5	10.9 ± 9.6	10.3 ± 9.6
Esophagus	D_max_ (Gy)	17.6 ± 9.7	16.9 ± 8.9	19.6 ± 10.6	19.3 ± 10.3

**Figure 1:**
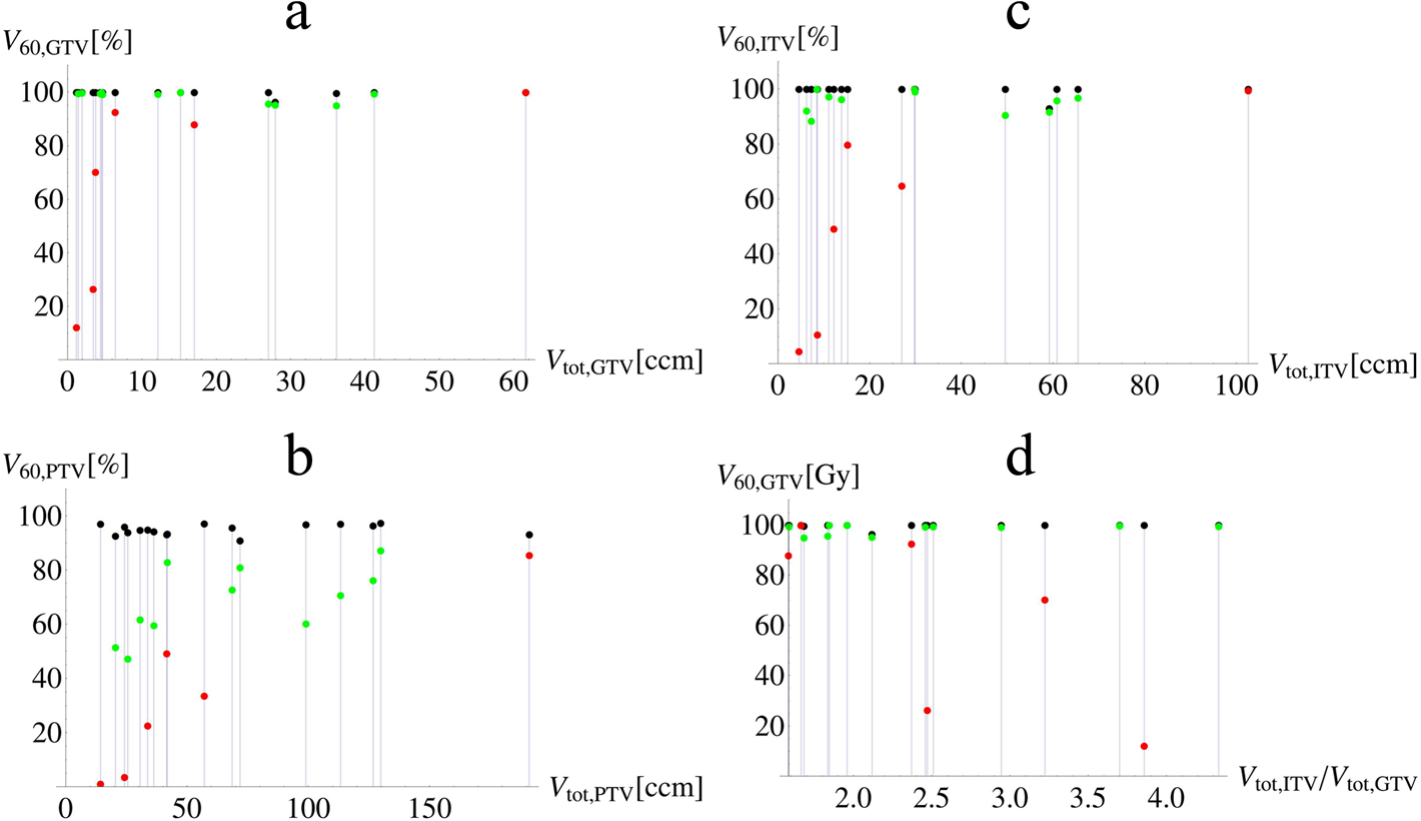
**Target coverage versus target volume for a) gross tumor volume (GTV), b) ITV and c) planning target volume (PTV); d) shows GTV coverage versus the ratio of ITV and GTV volume which is indicative of tumor movement.** The black markers indicate the original values calculated with pencil beam (PB), while the red and green markers show the recalculated collapsed cone (CC_re-calc_) patients with central tumors (red) and tumors attached to mediastinum or pleura (green).

**Figure 2:**
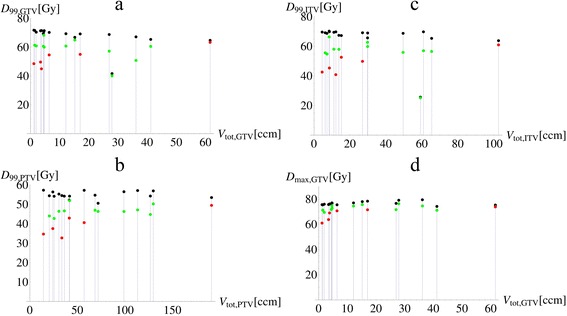
**D**_**99**_ **versus the total absolute volume for a) GTV, b) ITV and c) PTV; d) shows the maximal dose versus the total GTV volume.** The black markers indicate the original values calculated with pencil beam (PB), while the red and green markers show the recalculated collapsed cone (CC_re-calc_) patients with central tumors (red) and tumors attached to mediastinum or pleura (green).

The GTV, ITV and PTV coverage with 60 Gy and higher as well as D_99_ and D_max_ versus the GTV, ITV and PTV volume for both PB and CC_re-calc_ treatment plans are shown in Figures 1 and 2. For the PTV, a larger discrepancy between PB and CC coverage is significantly correlated with a smaller ROI volume (ρ_PTV_ = −0.77, p_PTV_ < 0.001), while for the GTV and ITV the correlation coefficient is less pronounced and not significant (ρ_ITV_ = −0.43, p_PTV_ = 0.08, ρ_GTV_ = −0.18, p_GTV_ = 0.49). Large difference of D_min_, D_99,_ D_max_ and D_1_ between PB and CC is significantly correlated with ROI volume for all three target ROIs: GTV, ITV and PTV.

There was considerable variability of the dose volume parameters of GTV, ITV and PTV between patients, likely due to tumor volume and position. As expected the differences were more pronounced for tumors completely surrounded by lung tissue and not adherent to pleural or mediastinum tissues. For example, the difference in average GTV relative volume covered by 60 Gy or more was 3.5% for peripheral tumors compared to 6.1% for centrally located tumors. Presumably, this is due to the fact that the density of the chest wall and mediastinal tissue is closer to the density of water, than the low density lung tissue. Therefore the PB algorithm, which assumes water density laterally, estimates dose more accurately in tumors partially attached to denser tissue than in those surrounded mainly by air.

**Figure 3:**
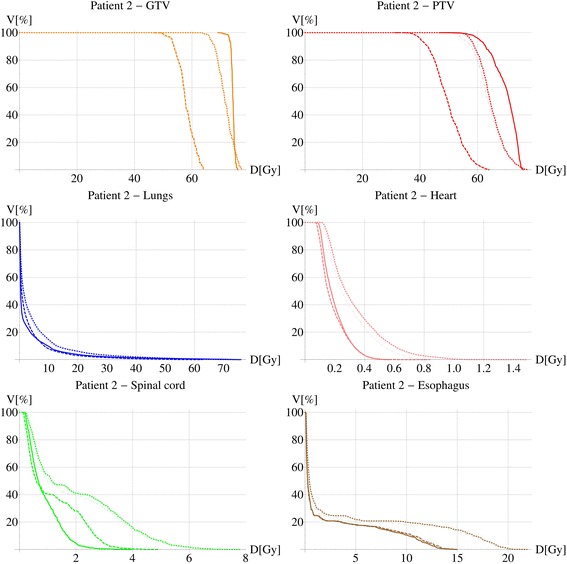
**Dose volume histograms of GTV, PTV, lungs, heart, spinal cord and esophagus for a patient with extreme differences between PB and CC distributions.** The solid, dashed, dotted and dashed-dotted lines represent the PB, CC_re-calc_, CC_cov_ and CC_clin_ plans respectively.

**Figure 4:**
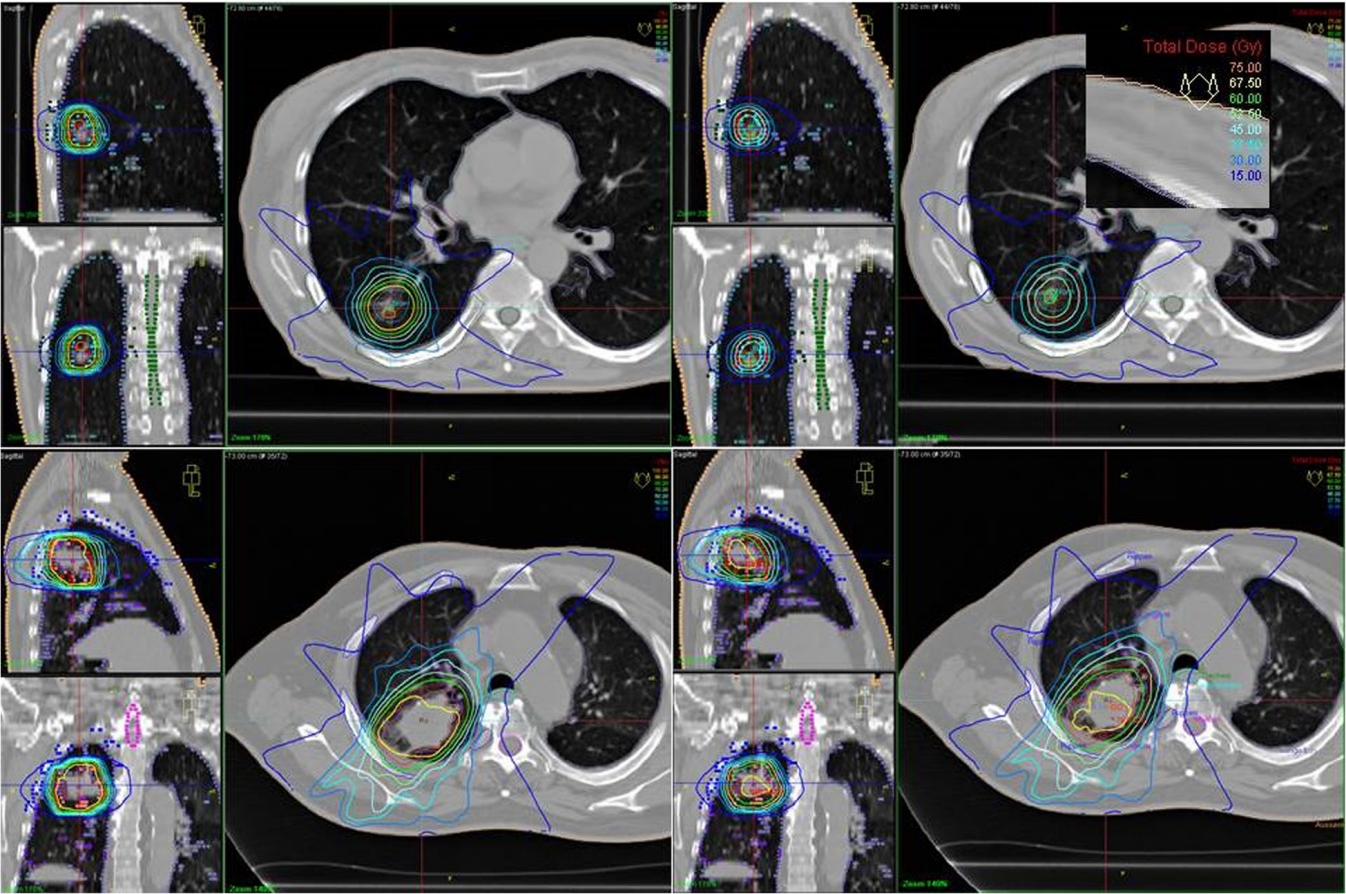
**Sagittal, coronal and transversal CT view of two sample patients.** The top slices show the most extreme case (Patient 2) due to the small tumor volume, whereas the bottom slices depict a less extreme case. Pencil beam (PB) is shown on the left and the plans recalculated with collapsed cone (CC_re-calc_) on the right. The orange, yellow, bright green, light green, light blue, medium blue, turquoise and dark blue lines represent the 75 Gy, 67.5 Gy, 60 Gy, 52.5 Gy, 45 Gy, 37.5 Gy, 30 Gy and 15 Gy isodoses, respectively

In one extreme case of a central tumor with very small volume the GTV V_60Gy_ was reduced from 100% to 26%, D_mean_ from 74.2 Gy to 57.9 Gy and D_99_ from 71.6 Gy to 49.9 Gy. The PTV coverage was tremendously reduced from 96% to 3.6%. The DVHs of this patient are shown in Figure 3. Dose distributions of this patient and another less extreme case with larger tumor volume close to the posterior chest wall are shown in Figure 4.

No correlation was found between tumor movement (which was quantified by the ratio of ITV volume to GTV volume) and difference in GTV coverage (Figure 1 bottom, right, ρ = 0.12, p = 0.63). However, this result may be limited to our study and a more rigorous analysis is necessary to evaluate the impact of tumor motion on dose distribution in general, for example by performing a 4D dose calculation study similar to Guckenberger et al. [5].

### PB vs. CC_clin_ and CC_cov_

For the GTV there was no statistically significant difference in D_mean_, D_min_, D_max_, D_99_, D_1_ when comparing PB to the plan with similar coverage (CC_cov_). By design, there was no difference in coverage (V_60Gy_). For PTV, there was a significant difference in D_mean_, D_min_, D_99_ and D_1_, but not in terms of coverage and D_max_. In order to achieve similar coverage, MUs delivered and field size in X and Y direction had to be adjusted. Total MUs and average field size in x and y direction were significantly larger in the CC_clin_ and CC_cov_ plans (p < 0.001, Table 3). Fields were more often adjusted craniocaudally.

**Table 3:** **Relative change in average total monitor units with respect to PB plans and average field size**

	**PB**	**CC**_**cov**_	**CC**_**clin**_
Average relative change of total MU (%)	-	5.0 ± 5.6	5.8 ± 5.9
Average field size X (cm)	4.6 ± 1.3	5.0 ± 1.3	4.9 ± 1.3
Average field size Y (cm)	6.5 ± 1.9	7.2 ± 1.9	6.9 ± 2.0

In current clinical practice we use the enhanced CC algorithm to calculate dose, and the field size is allowed to increase up to a distance of 10 mm between the jaws and the outer PTV margin in BEV (compared to 5 mm in former plans calculated with PB) to achieve more adequate calculated GTV coverage. Therefore, the clinically accepted plans CC_clin_ had acceptable GTV coverage that was not different from coverage calculated with the original PB. However, CC_clin_ plans had decreased D_min_ and D_99_ for the GTV.

The field size is usually not increased enough to also achieve the same planned PTV coverage as with PB, especially in areas mainly consisting of air. Thus, by construction, PTV values do not reach values of the original PB plan for CC_clin_ plans.

All evaluated parameters for the OAR were significantly larger in the CC_cov_ plan than PB and consequently in CC_re-calc_. This is due to the increased monitor units and field sizes necessary to achieve similar calculated target coverage for the CC_cov_ plan compared to the PB plan.

The results for the CC_cov_ and CC_re-calc_ plans imply that when switching dose calculation algorithms from PB to CC without changing the criteria for an acceptable treatment plan, the OAR will be exposed to increased dose. This is because when using CC the same plan quality in terms of reported target coverage (especially in the PTV) can only be achieved by increasing MU and field size, which leads to higher dose to OAR. Although the CC calculated dose is closer to the truly delivered dose and thus preferable over PB, many OAR constraints originate from experience with PB. While some of the available recommendations for lung SBRT may suggest adaptation of the dose prescription according to the type of dose-calculation algorithm used that is not nearly always the case for clinical recommendations [6].

The difference between dose estimates by using PB and CC in target structures is especially pronounced in the PTV, because the PTV contains tumor tissue as well as air in the planning CT and thus includes large variation of tissue density. Our data is in the range of previously reported works by Haedinger et al. [7], who found a decrease in D_mean_ of 11.2% whereas 14.2% was found in our study. However, we found a much more drastic change in average PTV coverage of 39.4 volume % compared to 7.1 volume % found by Haedinger et al., who used the Helax-TMS treatment planning system. It is therefore essential to keep stressing the importance of the choice of suitable dose calculation algorithms. Although there are a variety of studies that have investigated implications of using PB type versus CC type dose calculation algorithms (e.g. [7,8]), most publications on the matter consider the PTV and CTV mainly or exclusively. However, Guckenberger et al. [5] have shown that the 4D dose calculated over all breathing phases in the GTV is similar to the dose in the GTV in one single phase for 3D conformal plans (end-exhalation, end-inhalation, or mid-ventilation phase). This means that the GTV may really be the relevant ROI for evaluating such differences resulting from dose calculation algorithms assuming that the fields are opened enough to allow coverage of the GTV in all phases. The present study therefore also includes data for the GTV.

Aarhup and Dobler [3,4] demonstrated discrepancies in mixed-density phantom studies. They showed that PB algorithms tend to overestimate the target dose, while CC and MC seemed to provide more reliable data compared to measurements. Latifi et al. [9] demonstrated that there was a significantly higher rate of reoccurrence when SABR plans were planned using a PB algorithm for dose calculation than if CC was used.

The performance of dose calculation algorithms in lung depends on the use of different patient models [10] in order to account for target motion in the presence of large density inhomogeneities (i.e. one phase static CTs, average CTs, CTs with density overrides, maximum intensity projection) [11,12]. For the purpose of this study, we performed dose calculation only on a single static planning CT, which is the current clinical practice in our institution for lung SABR treatment planning using Oncentra MasterPlan®. Furthermore, it should be noted that both the quality of the beam model and the TPS-specific implementation of the head model can influence the performance of different dose calculation algorithms and impact the calculated dose distribution. The results obtained in this study may therefore be specifically useful for OTP users. For the purpose of our study we used PB- and CC-beam models that were carefully verified with respect to base data measurements prior to clinical use in our institution.

The large discrepancies resulting from use of different dose calculation algorithms are of special importance when multi-institutional studies are performed. A comparative planning study for the JCOG 0403 protocol showed notable differences between the participating institutions for D_max_, D_min_, D_95_, and the homogeneity index of the PTV, although target definitions and target dose constraints were the same. These inter-institutional deviations were mainly attributed to the different choice of dose calculation algorithms used in the institutions [13]. Even if only CC algorithms are used, their quality may depend on the exact implementation of the algorithm in the treatment planning system, and be specific to the release version [14,15]. The use of a wide variety of dose prescription modes in stereotactic radiotherapy leads to additional in-transparency when comparing data [16].

More recently started studies require tissue density heterogeneity correction. For RTOG 0236, SBRT conformal treatment plans were generated using XiO/superposition meeting dosimetric compliance criteria recommended for RTOG 0813 and recalculated using MC. Tissue density heterogeneity correction was applied in the initial calculations. V20Gy increased on average by 18% in the MC plans [17]. Although CC algorithms predict dose more accurately than PB algorithms, they can nevertheless deviate from measured dose and dose calculated with MC. Several studies investigated deviations of CC algorithms from measured dose or dose calculated with MC (e.g. [2,15,18]). Krieger et al. [2] and Kry et al. [18] found good agreement between MC and measured dose. Thus, in order to fully evaluate the implications of using PB and CC algorithms in the clinic, a comparison with MC would be necessary.

## Conclusions

The use of different calculation algorithms leads to significant changes not only in dose, but also in field size and MUs delivered, if similar target coverage criteria are applied for treatment planning. This has to be taken into account for treatment planning and comparison of data concerning side effects as well as local control in radiotherapy of intrapulmonary lesions. Using CC improves the accuracy of dose calculation in the tumor. However, attempting to cover the parts of the PTV and ITV that mainly consist of air, caused a higher strain on the lung and OARs in our study. In cases with borderline acceptable OAR exposition, CC plans should be evaluated cautiously.

If data concerning safety margins, OAR limits, maximum or mean dose and PTV/ITV coverage of radiotherapy plans in stereotactic radiotherapy are compared, it is of interest to state which dose calculation algorithm was used. Future projects will include comparison of the resulting CC and PB dose distributions to the MC gold standard, as dose calculated with CC itself can deviate from measured values. Also evaluation of accumulated 4D dose may be necessary to estimate the actual increase of TCP that can be achieved by trying to obtain PTV coverage with CC.

## Abbreviations

CC: Collapsed cone
PB: Pencil beam
MC: Monte Carlo
OAR: Organs at risk
SABR: Stereotactic ablative radiotherapy
DVH: Dose volume histogram
PTV: Planning target volume
IT: Internal target volume
CTV: Clinical target volume
GTV: Gross tumor volume
MU: Monitor unit
ROI: Region of interest
MLC: Multi leaf collimator
TCP: Tumor control probability
BEV: Beam’s eye view
TPS: Treatment planning system
